# Emotional Incontinence: A Case Report of Pseudobulbar Affect in the Setting of Alcohol Use Disorder

**DOI:** 10.7759/cureus.38976

**Published:** 2023-05-13

**Authors:** Christian Nwabueze, Muhammad Azam, Victor Kekere, Nkolika Odenigbo, Fahima Banu, Patrice Fouron

**Affiliations:** 1 Department of Psychiatry, Interfaith Medical Center, Brooklyn, USA

**Keywords:** pathophysiology, neuropsychiatric, alcohol use disorder, pseudobulbar palsy, emotional incontinence

## Abstract

Pseudobulbar affect (PBA) manifests as a disconnect between emotional feelings and emotional expressions. The impact of pseudobulbar affect on social, occupational, and interpersonal functioning is substantial. It results in poor quality of social interactions and poor overall quality of life. Instances of pseudobulbar affect with no underlying neuropsychiatric disorders are rarely reported in the literature. Although alcohol use has been associated with traumatic brain injuries (TBI), alcohol as a direct cause of pseudobulbar palsy has rarely been reported.

Our case presents a unique situation with no known underlying primary neurologic disorder but evidence from clinical history, physical examination, and laboratory tests indicative of severe alcohol use disorder. This case represents the rare instances where the disease etiology is unusual and reminds the health care provider to consider the role of alcohol in the pathophysiology of pseudobulbar affect. More research is needed to understand the role of alcohol in the etiology of pseudobulbar affect in the absence of any known underlying neuropsychiatric disorder.

## Introduction

Pseudobulbar affect (PBA) is a neuropsychiatric disorder that is socially debilitating [1]. It is characterized by unstimulated, uncontrolled, and exaggerated emotions such as laughing or crying, even in situations that do not warrant the behavior [2]. The episodes are unpredictable, involuntary, and often mood-incongruent [3]. The behavior may be disproportionate and inappropriate to the social context, indicating a disconnect between the patients’ expressed emotions and their experienced emotions [4]. Depending on preference, the nomenclature is diverse and includes emotional incontinence, emotionalism, emotional dysregulation, involuntary expression disorder, pathologic laughing and crying, and affective lability [3]. Pseudobulbar affect is usually secondary to a primary neurologic disorder or injury [1]. It is consequent to common neurologic disorders including multiple sclerosis (MS), amyotrophic lateral sclerosis (ALS), progressive supranuclear palsy, Parkinson’s disease, multiple system atrophy, dementias, brain tumors, Alzheimer’s disease, stroke, and traumatic brain injuries (TBI) [4]. The social, psychological, and emotional implications of PBA are significant [4]. They increase the burden of the disease on patients who are already suffering the debilitating effects of their underlying disorders [4]. It causes embarrassment for the patients, their caregivers, and their family and reduces the quality of social interactions [4]. This negatively affects the overall quality of health [4], including effects on occupational functioning, social functioning, and the quality of relationships [5]. Patients are often bothered and preoccupied by the likelihood of symptom reoccurrence [6].

Pseudobulbar affect is an important health issue in the United States, with an estimated upper limit of 7.1 million Americans affected by the disorder [4]. PBA is usually described in relation to underlying neurologic diseases, and the prevalence ranges from 5% to 50% depending on the study population and the methodologies and diagnostic criteria applied in the study [4]. The prevalence of PBA depends on the cause of the neurologic damage leading to the disorder [7]. Falconer et al. estimated that 26% of Parkinson’s disease patients have PBA [8] and 30%-35% are depressed [4]. The prevalence of PBA appears to increase with increasing age, is more prevalent among patients 50 years and older, and has been found to be higher among males compared to females among all age groups [7].

PBA may result from disorders of the frontal lobe with associated dysregulation and disinhibition [9]. Although the exact cause of PBA is unknown, it is probably related to disturbances in the limbic and paralimbic neural network systems [5]. The most widely acceptable theory indicates a disruption of the corticopontine-cerebellar circuit involved in modulating emotional responses [1]. Pseudobulbar affect has been linked to TBI [4], and although no direct relationship between alcohol use and PBA has been reported, alcohol use is significantly associated with TBI [10]. Most studies estimate that between 30% and 50% of patients treated for TBI were intoxicated at the time of injury [10].

The case we present demonstrated multiple episodes of alcohol intoxication with associated soft tissue trauma of the head, but no documented clinical or radiologic evidence of traumatic brain injury, stroke, multiple sclerosis, or any other neurologic disorders associated with PBA. It is, therefore, critical to investigate the role of alcohol in the occurrence of PBA, particularly in cases where the underlying neurologic causes are not evident.

## Case presentation

We present the case of a 66-year-old African American female with no past psychiatric history and a reported medical history of chronic obstructive pulmonary disease (COPD) and gallstone pancreatitis with co-occurring alcohol use disorder. She was admitted to the medical unit for vomiting and fatigue in the context of alcohol intoxication. She had already made multiple visits to the emergency room over a five-year period due to alcohol intoxication.

**Figure 1:**
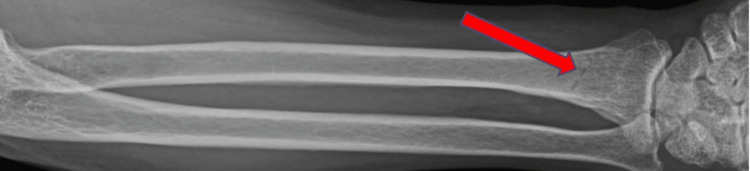
X-ray of a radial fracture from a fall during alcohol intoxication.

The patient first presented to the center about five years prior to the current hospitalization, when she was brought into the medical emergency unit for pain in the right arm due to a fall from height on an outstretched hand while intoxicated with alcohol. During the presentation, there was no loss of consciousness and no evidence of pseudobulbar palsy. Reports of X-rays done at that time revealed a non-displaced oblique fracture through the distal shaft of the radius (Figure 1). The fracture was splinted under local anesthesia, and the patient was discharged home after prolonged observation.

**Figure 2:**
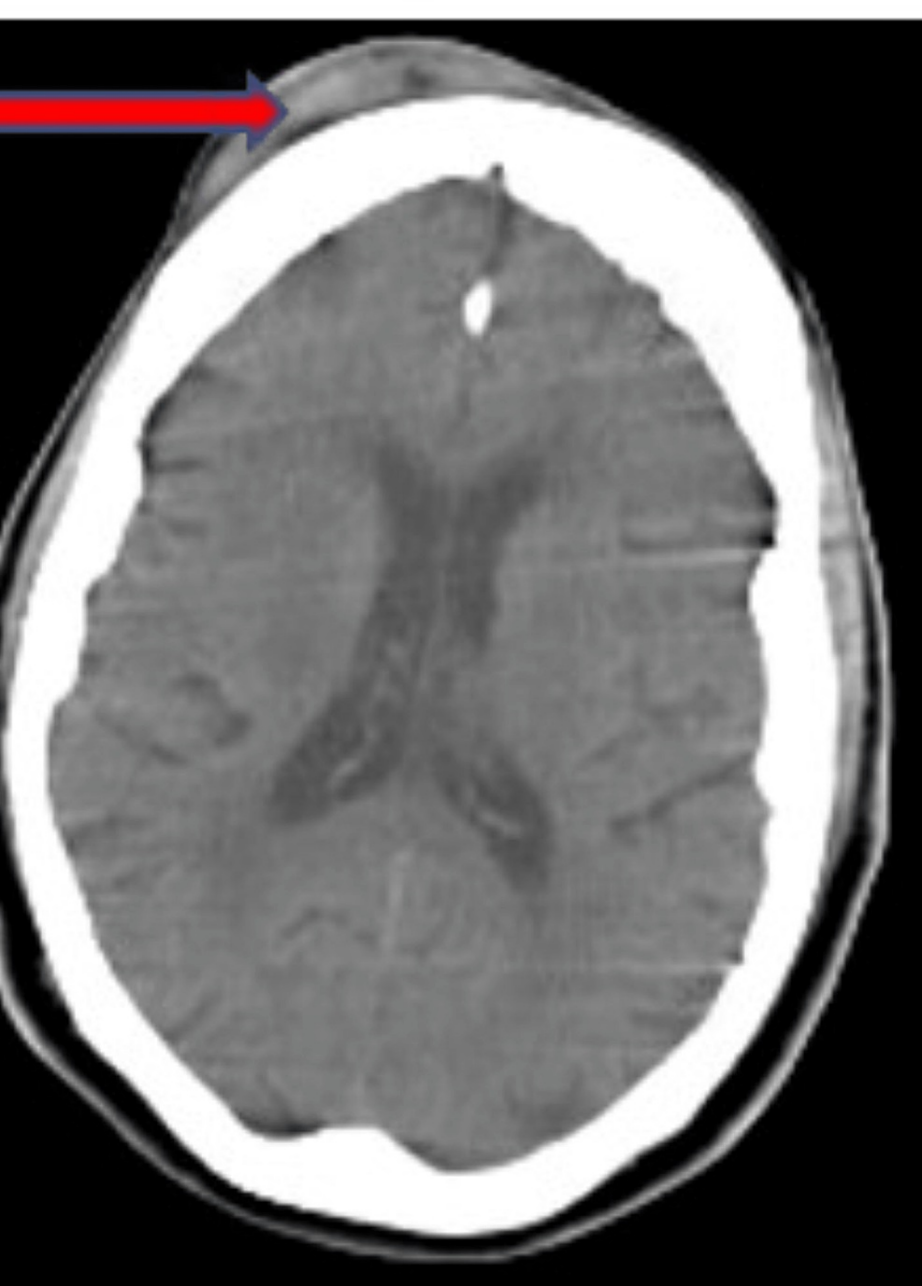
Head computed tomography scan without contrast showing a soft tissue hematoma in the right frontal location.

About two years after the initial fall, the patient again presented to the medical emergency unit with altered mental status following a fall while intoxicated with alcohol. Her family members reported that they found her lying on the sidewalk bleeding from the right side of the forehead, but they do not know how long she had been there or if she lost consciousness at any time. She was smelling of alcohol and reported that she had been drinking alcohol all day. She was conscious during the examination, and her Glasgow Coma Scale was 15. A computed tomography (CT) scan of the head without contrast (Figure 2) showed a soft tissue hematoma in the right frontal location, with no evidence of an acute bleed, traumatic brain injury, or infarct. The patient was monitored with serial examinations for several hours, and she became sober over many hours with stable mental status. She demonstrated the ability to ambulate safely, tolerated oral intake, and was discharged home.

About one year later, the patient presented again to the medical emergency unit complaining of generalized weakness after prolonged intake of alcohol, including a cup of vodka on the night of the presentation. She smelt of alcohol, was emotionally labile, and had slurred speech. The complete blood count (CBC) showed evidence of chronic alcohol use (Table 1), including megalocytosis and stomatocytosis. The patient was treated with intravenous fluid and intravenous thiamin. She was observed for about six hours and was discharged home after she became sober. She was counseled to decrease her alcohol intake and to follow up with her primary care doctor.

**Table 1:** Red blood cell evidence of chronic alcohol use. MCH: mean corpuscular hemoglobin, MCV: mean corpuscular volume.

Blood parameter	Laboratory findings	Status	Normal range
MCV	108.5	Abnormal	83–94.5 fL
MCH	36.9	Abnormal	26.9–32.5 pg
Hypochromasia	1+	Abnormal	None
Microcytosis	1+	Abnormal	None
Stomatocytes	1+	Abnormal	None
Teardrop cells	1+	Abnormal	None

**Figure 3:**
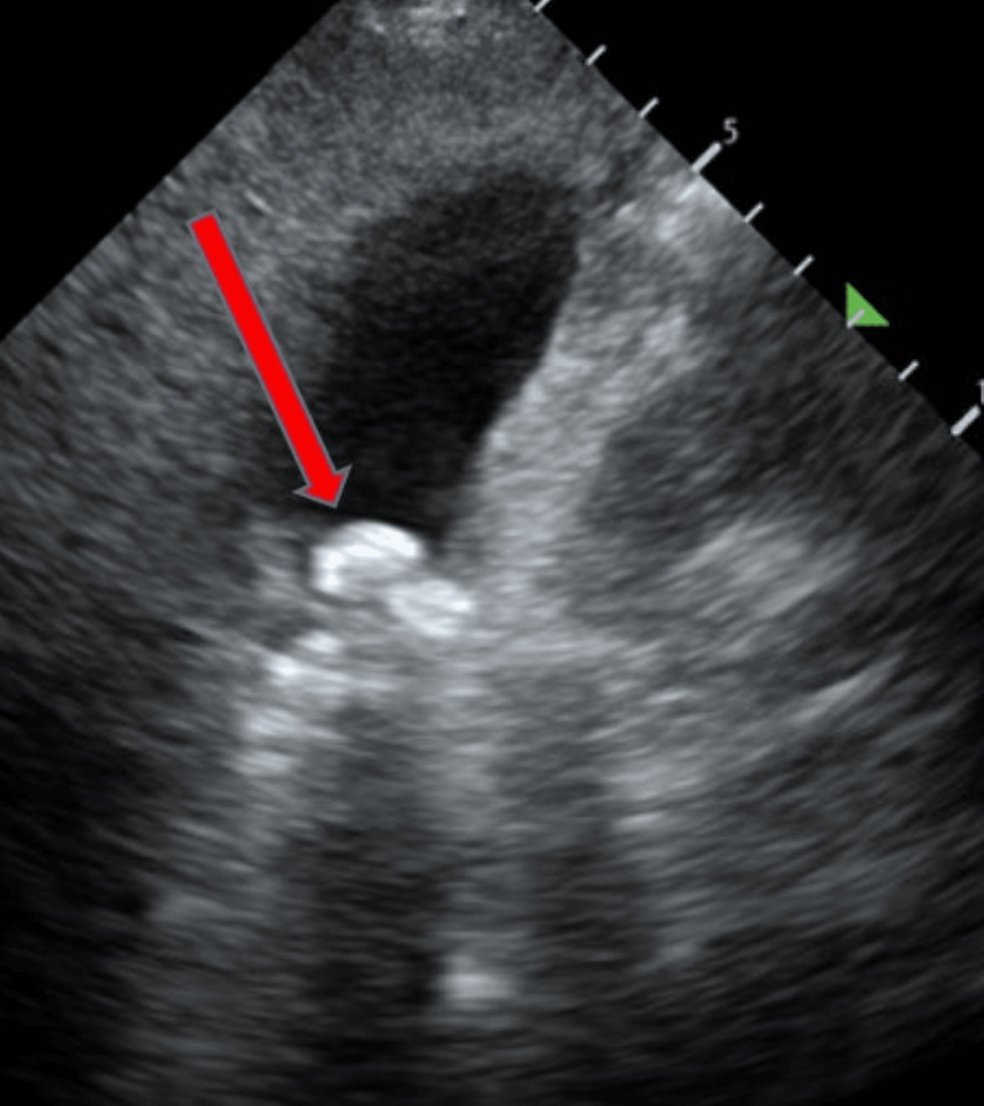
Ultrasound scan of the gallbladder showing multiple gallstones.

During her most recent hospitalization in 2022 (about five years after the initial contact), she was admitted to the intensive care unit (ICU) for acute gallstone pancreatitis following complaints of several days of malaise and multiple episodes of vomiting. She was toxic-appearing and obviously intoxicated. Her blood alcohol level at this presentation was 202 mg/dL (normal range, 0.00-10.0). A comprehensive metabolic panel (CMP) revealed multiple abnormalities, including elevated bilirubin and liver enzymes. The magnesium level was 1.1 mg/dL, serum lipase was >900. Measurement of serum vitamin B12 level was 640 pg/dL (reference range: 160-950 pg/mL) and serum folate level was 2.63 nmol/L (reference range: 4.5-45 nmol/L). A serum thiamin level was not obtained during the hospitalization. A bedside ultrasound scan revealed gallstones (Figure 3).

Assessment for alcohol use revealed that the patient has severe alcohol use disorder (Table 2). She reported tolerance to alcohol, deriving fewer highs and satisfaction from usual quantities of alcohol. She craved alcohol after a few hours of not using it, and she never attempted to cut it down. Because of her cravings and tolerance, she used more than she intended. She spent significant time obtaining, using, and recovering from the effects of her alcohol use. Despite her health problems and her previous alcohol intoxication, she continued to use large quantities of alcohol. She had problems with her family due to her unrelenting alcohol use.

**Table 2:** Screening for alcohol use disorder in the patient.

Symptoms of alcohol use disorder	Patient’s response	Score
Tolerance to alcohol	Yes	1
Alcohol withdrawal	No	0
Craving to use alcohol	Yes	1
Using more alcohol than intended	Yes	1
Unable to reduce or stop using alcohol	No	0
Spending significant time obtaining, using, and recovering from alcohol use	Yes	1
Use despite acknowledging health problems	Yes	1
Using despite social occupational and other adverse consequences of alcohol use	Yes	1
Neglecting other responsibilities because of alcohol use	No	0
Neglecting other activities because of alcohol use	No	0
Risky or dangerous behaviors or situations because of alcohol use	No	0
Total score		6

A psychiatry consultation was requested due to the patient's involuntary crying and laughing. The patient reported involuntary crying and laughing that had been occurring for two years but was not present during her last hospitalization for alcohol intoxication. She reported that the episodes were sudden and brief. She stated that she does not understand why she cries and laughs uncontrollably and that other people have also observed that she laughed and cried involuntarily. She reported feeling embarrassed by these episodes. She denies any past history of psychiatric illness, including depression. There were no symptoms suggestive of major depressive disorder. The patient denied anhedonia, changes in appetite, sleep, or concentration. She denied feeling excessive guilt, denied suicidal or homicidal ideation, intent, or plan. She denied auditory or visual hallucinations. 

On evaluation, the patient was observed having multiple episodes of crying and laughing that were sudden and involuntary. Her thought process was linear, logical, and goal-directed. Her thought content was devoid of paranoid or any other form of delusions. We assessed her using the Center for Neurologic Study-Laughing Scale (CNS-LS) for pseudobulbar affect. The CNS-LS is a short (seven-item), self-administered questionnaire designed to be completed by the patient that provides a quantitative measure of the perceived frequency of PBA episodes. A CNS-LS score of 13 or higher may suggest PBA [11,12]. The patient had a score of 28 out of 35, indicating a positive score. The patient also scored 29 out of 54 in the Pathological and Crying Scale (PLCS) interview, further strengthening the suspicion. A CT scan of the head without contrast showed brain volume loss, chronic small vessel ischemic disease, and mildly enlarged ventricles. These findings were compatible with her age and alcohol use disorder. There was also evidence of a chronic medial right orbital wall fracture, most likely from prior falls, and no evidence of traumatic brain injury.

## Discussion

Our patient represents a unique case of pseudobulbar affect in the absence of any known neurologic cause of the disorder. Pseudobulbar palsy is associated with traumatic brain injury, underlying Parkinson’s disease, Alzheimer’s disease, brain tumors, and other neurologic disorders [4]. These neurologic disorders were not reported in our patient. Episodes of sudden, involuntary crying and laughing are usually evidence of an underlying primary neurologic disorder [1]. Many neurologic disorders have been described in these individuals with PBA, including underlying Parkinson’s disease, Alzheimer’s disease, brain tumors, and traumatic brain injuries [4]. There is a paucity of literature in which patients present with PBA without an underlying primary neurologic disorder.

In the case we have presented, there was no clinical or laboratory evidence of an underlying neurologic condition. Even though our patient has had multiple falls, none has resulted in documented loss of consciousness, clinical or radiologic evidence concerning for TBI [13]. There is, however, ample evidence of an alcohol use disorder in our patient. Without a documented neurologic etiology for the PBA in our patient, we consider the neurologic damage of alcohol on the brain as a possible etiology [14]. Alcohol disrupts communication pathways in the brain causing alterations in neuronal sizes, appearances, and functions [15]. The neurotoxic actions of alcohol are responsible for the neurologic complications related to alcohol use [13]. Cognitive deficits, including changes in memory, attention, executive functions, and visuospatial functioning [16], as well as problems with balance due to disruption of cerebellar-cortical functional connectivity [17], have all been reported with alcohol use. The patient presented with features of PBA without documented clinical evidence of any underlying neurologic disorder. This finding corroborates the need for clinicians to investigate other non-traditional causes as possible etiologies of PBA. Further research is needed to establish any direct role of alcohol use disorder in the etiology of pseudobulbar palsy in the absence of currently established causes of the disorder.

## Conclusions

There is no evidence in our patient to indicate any underlying neurologic disorder. What is evident from the patient’s history, physical examination, and laboratory findings is a pattern of chronic alcohol use with a resultant alcohol use disorder. The extent of alcohol use by our patient is also indicated by the blood alcohol levels during some of her presentations and the red blood cell evidence of chronic alcohol use, including stomatocytosis.

In a patient such as ours where no neurologic evidence of PBA is found, the toxic effects of alcohol use on the neural cells, particularly at the microscopic level, deserve to be investigated. It is therefore imperative for clinical care providers to consider alcohol use disorder as a possible etiology and to obtain alcohol use history and evaluate the extent of alcohol use disorder in patients with PBA. Even in patients with no known underlying primary disorder, the toxic neurologic effects of chronic alcohol use on the brain may be implicated in the development of PBA.
